# APOLLO, a testis-specific *Drosophila* ortholog of importin-4, mediates the loading of protamine-like protein Mst77F into sperm chromatin

**DOI:** 10.1016/j.jbc.2023.105212

**Published:** 2023-09-02

**Authors:** Alexander V. Emelyanov, Daniel Barcenilla-Merino, Benjamin Loppin, Dmitry V. Fyodorov

**Affiliations:** 1 Department of Cell Biology, Albert Einstein College of Medicine, Bronx, New York, USA; 2 Laboratoire de Biologie et Modélisation de la Cellule, École Normale Supérieure de Lyon, CNRS UMR5239, Université Claude Bernard Lyon 1, Lyon, France

**Keywords:** spermatogenesis, chromatin remodeling, histone chaperone, sperm chromatin, sperm nuclear basic protein (SNBP), mst77F, importin-4, Artemis, Apollo

## Abstract

DNA in sperm is packed with small, charged proteins termed SNBPs (sperm nuclear basic proteins), including mammalian and *Drosophila* protamines. During spermiogenesis, somatic-type chromatin is taken apart and replaced with sperm chromatin in a multistep process leading to an extraordinary condensation of the genome. During fertilization, the ova face a similarly challenging task of SNBP eviction and reassembly of nucleosome-based chromatin. Despite its importance for the animal life cycle, sperm chromatin metabolism, including the biochemical machinery mediating the mutual replacement of histones and SNBPs, remains poorly studied. In *Drosophila*, Mst77F is one of the first SNBPs loaded into the spermatid nuclei. It persists in mature spermatozoa and is essential for sperm compaction and male fertility. Here, by using *in vitro* biochemical assays, we identify chaperones that can mediate the eviction and loading of Mst77F on DNA, thus facilitating the interconversions of chromatin forms in the male gamete. Unlike NAP1 and TAP/p32 chaperones that disassemble Mst77F–DNA complexes, ARTEMIS and APOLLO, orthologs of mammalian importin-4 (IPO4), mediate the deposition of Mst77F on DNA or oligonucleosome templates, accompanied by the dissociation of histone–DNA complexes. *In vivo*, a mutation of testis-specific *Apollo* brings about a defect of Mst77F loading, abnormal sperm morphology, and male infertility. We identify IPO4 ortholog APOLLO as a critical component of sperm chromatin assembly apparatus in *Drosophila*. We discover that in addition to recognized roles in protein traffic, a nuclear transport receptor (IPO4) can function directly in chromatin remodeling as a dual, histone- and SNBP-specific, chaperone.

In many animals, sperm DNA is tightly packaged with sperm nuclear basic proteins (SNBPs) that replace nucleosomes during spermiogenesis, the differentiation of haploid spermatids. While mammalian protamines are the best characterized SNBPs, *Drosophila* is a powerful alternative model to study the histone-to-protamine transition. *Drosophila melanogaster* sperm chromatin comprises several highly basic, protamine-like SNBPs that all share an MST-HMG-Box motif (1, 2, 3). Genetic interaction analyses have revealed that major *Drosophila* SNBPs, such as protamines (ProtA and ProtB), Prtl99C and male-specific transcript 77F (Mst77F), generally cooperate to achieve proper sperm chromatin organization (4, 5, 6). In this context, Mst77F is essential for male fertility, and post-transition spermatids eventually degenerate in *mst77F* mutant males (6). How SNBP deposition is controlled at the histone-to-protamine transition remains poorly understood.

Karyopherins (importins) are a superfamily of conserved receptor proteins that mediate the post-synthetic transport of large (>50 kDa) nuclear proteins from the cytoplasm across the nuclear pore complex into the nucleus (7, 8). According to a conventional model, importins α (adaptor proteins) recognize and bind a nuclear localization signal within their cargo molecule (9), recruit importins β (nuclear transport receptors, NTRs), and the trimeric complex permeates the nuclear pore complex by means of interactions between importin β and FG repeats of nucleoporins (10, 11). After the importin–cargo complex reaches the nucleus, binding and the enzymatic activity of Ras-related nuclear protein–GTP complex (RanGTP) control the release of cargo proteins (8) and determine the direction of nucleocytoplasmic transport of dissociated importins (12, 13). Recent evidence also suggests that most importins β/NTRs bind cargo directly and do not depend on adaptors (importins α) for nuclear transport (12, 14). The importin β family includes more than a dozen members (12, 14), such as importins 1 to 5, 7 to 9, 11, and 13, characterized by the presence of conserved functional domains: RanGTP-binding N-terminal domain (IBN_N) and karyopherin β domain (KAP95), interspersing several flexible HEAT repeats (15). The HEAT repeats are required for direct cargo binding by importins β, and their RanGTP-dependent conformational changes are thought to regulate the release of cargo proteins (13).

The *D. melanogaster* genome encompasses tandem duplicate genes *Apollo* (*Apl*) and *Artemis* (*Arts*), encoding nearly identical (∼98% identity, >99% similarity) proteins APOLLO/APL and ARTEMIS/ARTS, orthologs of a mammalian importin β, importin-4/IPO4 (16). Despite their structural relationship, APL and ARTS exhibit drastically distinct, sex-specific expression patterns, with *Apl* transcribed predominantly in the testis and *Arts* in the ovary. Their individual mutations, although not detrimental to adult development, lead to male or female sterility phenotypes (16), consistent with their expression patterns.

Upon entering *Drosophila* eggs, sperm chromatin undergoes remodeling to replace SNBPs and assemble nucleosomes (17). Biochemical evidence indicates that the removal of ProtA and ProtB is facilitated by a combined action of chaperone proteins nucleosome assembly protein 1 (NAP1), nucleoplasmin-like protein (NLP), nucleophosmin, and TAP/p32 (18), which can also function as core histone chaperones (19). In this study, we use a similar biochemical approach to identify molecular chaperones that can mediate the metabolism of Mst77F during interconversions of sperm and somatic-type chromatin when Mst77F is deposited in the genome (spermatogenesis) or evicted and replaced by histones (egg fertilization). We discover that in addition to NAP1, NLP, and TAP/p32, the IPO4 orthologs APL and ARTS also physically interact with Mst77F. Furthermore, APL mediates the replacement of core histones with Mst77F *in vitro* and is required for Mst77F deposition in *Drosophila* spermatids *in vivo*.

## Results

### Isolation of putative chaperones for Mst77F

**Figure 1:**
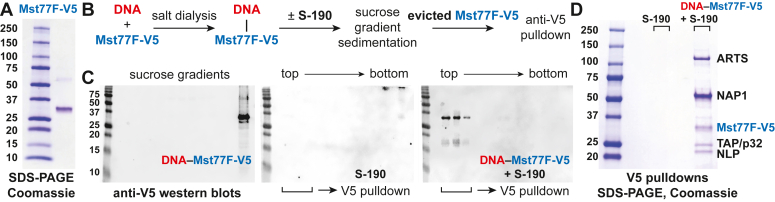
**Mst77F eviction activity and purification of putative Mst77F chaperones from *Drosophila* embryonic extract.***A*, SDS-PAGE of purified recombinant Mst77F-V5. ∼0.5 μg protein was loaded next to a molecular mass marker. Sizes (kDa) are shown on the *left*. *B*, schematic of the experiment to purify putative Mst77F chaperones (see Experimental Procedures). *C*, V5 immunoblot analyses of fractions from sucrose gradient sedimentation of the DNA–Mst77F-V5 substrate before and after treatment with the S-190 extract. Brackets at the *bottom* indicate fractions used for V5 pulldown experiments. Note the virtual lack of V5-immunoreactive material in the S-190-only sedimentation fractions. Molecular mass marker sizes (kDa) are shown on the *left*. *D*, SDS-PAGE of proteins that copurify with Mst77F-V5 evicted from the DNA–Mst77F-V5 substrate by treatment with S-190. Polypeptides were identified by mass-spec sequencing of excised gel bands (Fig. S1*A*). There are no detectable bands in pulldown experiments with S-190 in the absence of Mst77F-V5. Marker sizes (kDa) are shown on the *left*. ARTS, ARTEMIS; Mst77F, male-specific transcript 77F; NAP1, nucleosome assembly protein 1; TAP/p32, HIV-1 Tat-associated protein/p32.

To isolate chaperone molecules for Mst77F, we followed the approach previously utilized to identify chaperones for *Drosophila* ProtA and ProtB (18). We expressed and purified recombinant full-length Mst77F with a C-terminal V5 tag (Fig. 1*A*). The Mst77F–DNA complex was reconstituted by dialysis with a supercoiled plasmid and used as a substrate for remodeling by embryonic S-190 extract *in vitro* (Fig. 1*B*). The association of Mst77F-V5 with the plasmid DNA was assayed by sucrose gradient sedimentation and V5 western analyses of the gradient fractions (Fig. 1, *B* and *C*). Similar to protamine–DNA complexes (18), the Mst77F-DNA substrate sedimented to the bottom of the gradient, whereas upon treatment with S-190, Mst77F was released from its binding to DNA (Fig. 1*C*, *cf.* left and right panels). Since molecular chaperones that remove Mst77F from DNA likely remain associated with it upon eviction (18), we purified these putative protein complexes from gradient fractions that contained the released Mst77F by V5 immunoaffinity chromatography (Fig. 1, *B* and *C*) and analyzed them by SDS-PAGE (Fig. 1*D*). As a control, pulldown experiments were also performed with equivalent fractions from Mst77F-free, S-190-only sucrose gradients (Fig. 1*C*, middle panel, and Fig. 1*D*). The identities of purified polypeptides were established by mass-spec sequencing (Fig. S1*A*).

In addition to V5-tagged Mst77F, the immunoprecipitated proteins included NAP1, TAP/p32, and NLP, the histone chaperones that were previously implicated in the remodeling of ProtA/B-DNA substrates (18). Moreover, we also identified the protein ARTS, a *Drosophila* ortholog of IPO4. To confirm their physical interactions with Mst77F, we expressed recombinant ARTS, NAP1, NLP, and TAP/p32 (Fig. S1*B*) and examined them by co-immunoprecipitation (co-IP) with free Mst77F-V5 (Fig. S1*C*). We discovered that each individual polypeptide directly interacted with Mst77F in solution.

### Remodeling of DNA-Mst77F substrate by recombinant chaperones *in vitro*

**Figure 2:**
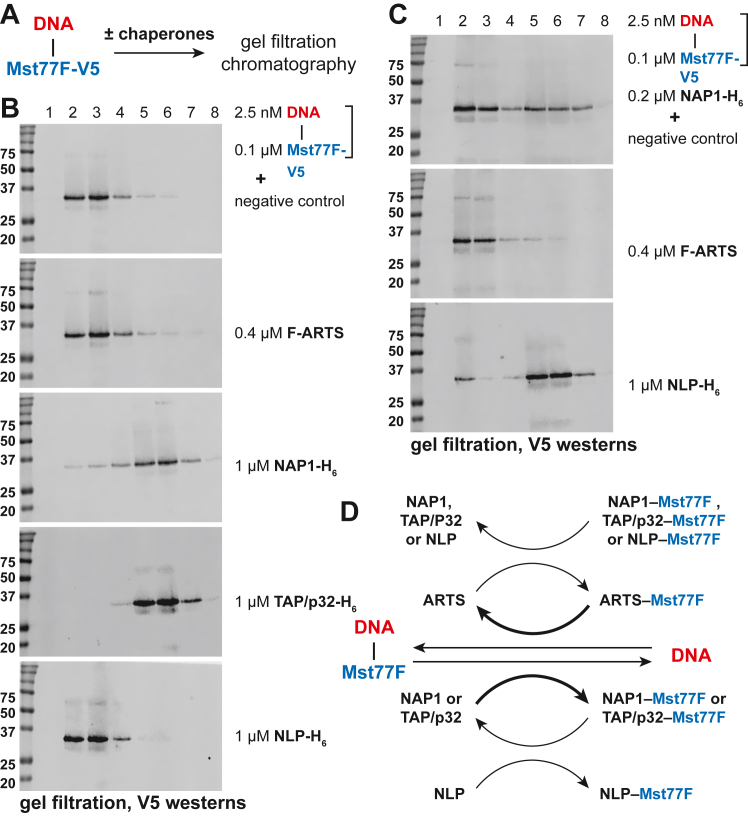
**Remodeling of DNA–Mst77F-V5 substrate by putative Mst77F chaperones *in vitro*.***A*, schematic of the *in vitro* experiment to examine the eviction of Mst77F from its complex with DNA by putative Mst77F chaperones. *B*, V5 immunoblot analyses of fractions from gel filtration of the DNA–Mst77F-V5 substrate before and after treatment with recombinant proteins (Fig. S1*B*). DNA–Mst77F-V5 substrate fractionates in “heavy” fractions (##2–3), whereas the released Mst77F-V5 fractionates in “light” fractions (##5–6). Protein and substrate concentrations in the reactions are shown on the right. Molecular mass marker sizes (kDa) are shown on the *left*. *C*, inhibition (by ARTS) or stimulation (by NLP) of the Mst77F eviction activity by substoichiometric NAP1. The analyses were performed and presented as in (*B*). Marker sizes (kDa) are shown on the *left*. *D*, the conversions of DNA–Mst77F-V5 substrate by Mst77F chaperones and protein–protein interactions in the *in vitro* system. NAP1 or TAP/p32 evicts Mst77F-V5 from its complex with DNA; Mst77F-V5 is transferred to NLP, thus releasing free NAP1 or TAP/p32 and facilitating further eviction of Mst77F-V5; when Mst77F-V5 is transferred to ARTS, it mediates the deposition of Mst77F-V5 back on DNA, thus counteracting the activity of NAP1 or TAP/p32. ARTS, ARTEMIS; Mst77F, male-specific transcript 77F; NAP1, nucleosome assembly protein 1; TAP/p32, HIV-1 Tat-associated protein/p32.

To further characterize the molecular functions of the identified Mst77F chaperones, we examined the activities of recombinant proteins in the eviction of Mst77F from its complex with DNA *in vitro* (Fig. 2*A*). In these experiments, we used size-exclusion chromatography to separate released Mst77F from the Mst77F–DNA complex (Fig. 2, *A*–*C*), instead of sucrose gradients (Fig. 1*C*), which are more suitable for large-scale bulk reactions. As expected, the Mst77F-DNA substrate alone fractionated in early (heavy) fractions (Fig. 2*B*, top). However, when treated with super-stoichiometric recombinant NAP1 or TAP/p32, the Mst77F–DNA complex was dissociated, and Mst77F fractionated in later (light) fractions (Fig. 2*B*). In contrast, ARTS and NLP alone failed to efficiently remodel the substrate.

This finding poses an apparent contradiction with the efficient binding of recombinant ARTS and NLP to Mst77F (Fig. S1*C*) and the discovery of native ARTS and NLP in association with Mst77F that was released from the Mst77F–DNA complex upon remodeling by S-190 (Fig. 1*D*). However, the contradiction may be resolved if, in the S-190 remodeling reactions, Mst77F is exchanged between NAP1 or TAP/p32 (the true eviction factors) and ARTS or NLP. For instance, when the eviction factors are limiting (substoichiometric to Mst77F), this exchange would liberate NAP1 and TAP/p32 for additional rounds of Mst77F eviction, thus stimulating the reaction. Indeed, sub-stoichiometric NAP1 remodels the Mst77F-DNA substrate inefficiently (*cf.* Fig. 2*C*, top, and Fig. 2*B*, middle). However, an excess of NLP in addition to NAP1 strongly stimulated the activity of NAP1 (*cf.* Fig. 2*C*, top and bottom).

In contrast, an excess of ARTS failed to stimulate the NAP1-mediated release of Mst77F, but, to the contrary, strongly inhibited the reaction (Fig. 2*C*, middle). It also partially reversed the near complete remodeling of the Mst77F–DNA substrate by super-stoichiometric NAP1 and TAP/p32 (*cf.* Figs. S2 and 2*B*). This result is consistent with the ability of ARTS to load Mst77F on the DNA, rather than release it from the Mst77F–DNA complex. The proposed molecular roles of NAP1, TAP/p32, ARTS, and NLP in the metabolism of the Mst77F–DNA complex are schematized in Figure 2*D*.

### Reconstitution of Mst77F loading on DNA *in vitro*

**Figure 3:**
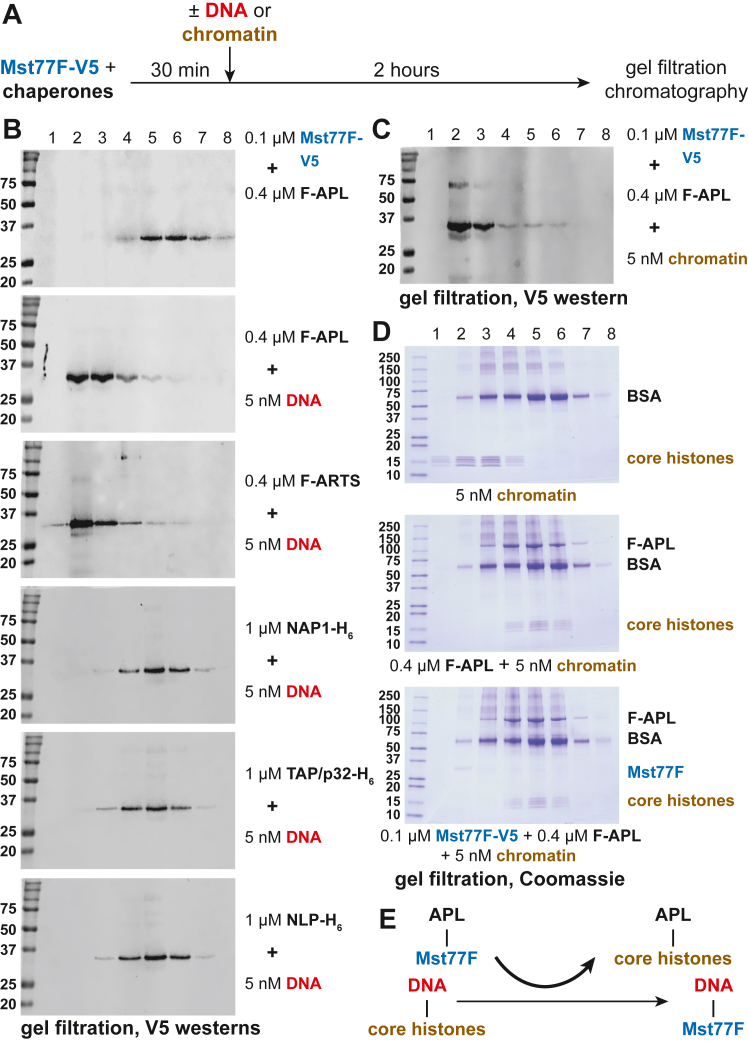
**APL-mediated deposition of Mst77F-V5 on DNA and chromatin *in vitro*.***A*, schematic of the *in vitro* experiment to examine the deposition of Mst77F on DNA or chromatin by putative Mst77F chaperones. *B*, V5 immunoblot analyses of fractions from gel filtration of the chaperone–Mst77F-V5 complexes before and after providing the DNA substrates for deposition of Mst77F-V5. Experimental results are presented as in Figure 2*B*. Molecular mass marker sizes (kDa) are shown on the *left*. *C*, Mst77F-V5 deposition on the oligonucleosomal substrate by APL *in vitro*. The experiment was performed as in (*A*) and presented as in (*B*). Marker sizes (kDa) are shown on the *left*. *D*, SDS-PAGE of fractions from gel filtration of the oligonucleosomal substrate before and after the removal of core histones by APL. Core histones in the assembled oligonucleosomes fractionate in “heavy” fractions (##1–4), whereas the evicted core histones fractionate in “light” fractions (##4–6). During chromatin disassembly by APL–Mst77F-V5 complex, Mst77F-V5 is deposited on DNA and fractionates in “heavy” fractions (##2–3); compare bottom panel to (*C*). Marker sizes (kDa) are shown on the *left*. *E*, the conversions between oligonucleosomes and DNA–Mst77F-V5 substrate by APL in the *in vitro* system. APL deposits Mst77F-V5 on DNA while simultaneously disassembling nucleosomes and releasing core histones. APL, APOLLO; ARTS, ARTEMIS; Mst77F, male-specific transcript 77F; NAP1, nucleosome assembly protein 1; TAP/p32, HIV-1 Tat-associated protein/p32.

*In vivo*, ARTS is primarily expressed in the ovary (16). Therefore, it is difficult to explain the physiological relevance of its putative role in the deposition of Mst77F on DNA, which occurs during spermatogenesis in the testis (6). As opposed to ARTS, its paralog APL, which is almost identical, is expressed in the male germline. To examine the possible activity of APL in Mst77F deposition on DNA, we expressed recombinant APL (Fig. S3*A*) and performed loading experiments *in vitro*. Briefly, Mst77F–APL complexes were incubated with plasmid DNA, and Mst77F association with the DNA was analyzed by size-exclusion chromatography and western blot of fractions (Fig. 3*A*). As anticipated, both APL and ARTS facilitated the assembly of the Mst77F–DNA complex *in vitro*, unlike NAP1, TAP/p32, or NLP (Fig. 3*B*). For instance, in the absence of an acceptor DNA template, APL cofractionated with Mst77F-V5 (Fig. S3*B*). However, upon addition of the plasmid DNA, Mst77F-V5 was loaded on the template, whereas APL remained in the low molecular mass column fractions (Fig. S3*B*).

This observation suggests that APL may mediate the deposition of Mst77F on DNA *in vivo*. Importantly, the physiological substrate of APL is a nucleoprotein complex, rather than naked DNA. Therefore, we examined the loading of Mst77F on the *in vitro* reconstituted oligonucleosome substrate and observed an efficient association of Mst77F with DNA/chromatin in the presence of APL (Fig. 3*C*). Furthermore, Mst77F deposition *in vivo* must be accompanied by the disassembly of chromatin and removal of core histones (and other chromatin components). It is possible that APL can mediate both the Mst77F deposition and core histone eviction. Indeed, when gel filtration fractions were analyzed by SDS-PAGE, we discovered that core histones were removed from oligonucleosomes by APL and cofractionated with it (Fig. 3*D*). The nucleosome disassembly did not depend on Mst77F and could be mediated by APL alone (Fig. 3*D*, middle panel).

Thus, *in vitro*, APL mediates the exchange of core histones for Mst77F (Fig. 3*E*). This mechanism indicates that APL also functions as a core histone chaperone. It further suggests that APL must exhibit a higher affinity toward core histones than toward Mst77F. To further confirm this idea, we examined the binding of APL to Mst77F and/or purified core histones by glycerol gradient sedimentation. Whereas both free Mst77F and core histones sedimented at the top of the gradient (Fig. S3*C*), substoichiometric APL interacted with a fraction of Mst77F and shifted its sedimentation toward the middle of the gradient (Fig. S3*D*). As predicted, core histones, when mixed with Mst77F, efficiently competed with it for binding to the limiting APL (Fig. S3*E*). This result strongly supports the proposed model for the function of APL in the concomitant disassembly of nucleosomes and assembly of Mst77F–DNA complexes (Fig. 3*E*).

### The functional roles of APL during spermatogenesis *in vivo*

**Figure 4:**
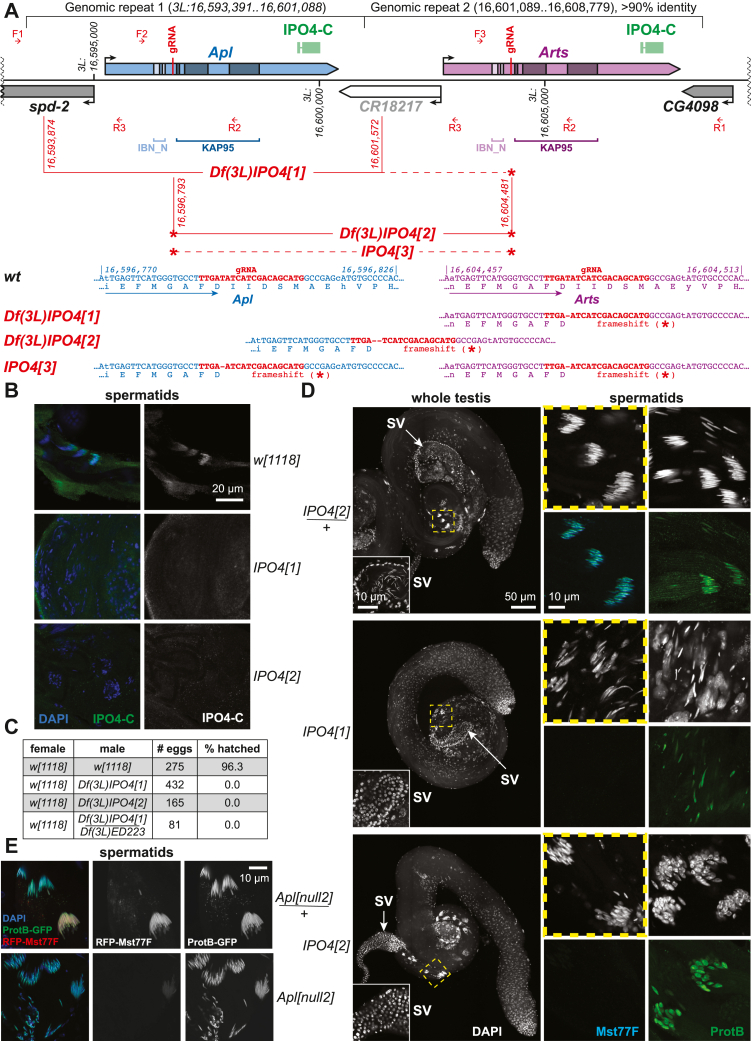
**APL-mediated deposition of Mst77F into sperm chromatin during spermatogenesis *in vivo*.***A*, *Apl–Arts* locus in the *Drosophila* genome, *Apl* and *Arts* mutant alleles and sequences for generating IPO4-C antibody. *Apl* gene and nucleotide/amino acid sequences are shown in *blue*; *Arts* gene and nucleotide/amino acid sequences are shown in *purple*; *lighter* and *darker color shades* represent exonic sequences coding for their respective conserved IBN_N and KAP95 domains; *red vertical lines* and sequences correspond to the guide RNA; 3L genomic coordinates (BDGP Release 6) are shown; *red brackets* designate deficiency break points; small CRISPR/Cas9-generated deletions (indels) that disrupt both *Apl* and *Arts* are indicated by *red asterisks*. *Small red arrows* (F1-F3 and R1-R3), diagnostic PCR primers. Nonconservative residues are shown in lower-case typeface in the sequences. *B*, confocal images of *Drosophila* spermatid nuclei stained with IPO4-C antibody. APL localizes in spermatid nuclei in *w[1118]* (control) testis but is not detected in the nuclei of homozygous mutant (*Df(3L)IPO4[1]* or *Df(3L)IPO4[2]*) spermatids. *Blue*, DAPI staining of DNA; *green or white*, IPO4-C IF staining; scale bar, 20 μm. *C*, embryo hatching ratio as a measure of male fertility. Eggs laid by *w[1118]* (control) females mated to homozygous *Df(3L)IPO4[1]* or *Df(3L)IPO4[2]* males completely fail to hatch into larvae. The male sterility phenotype is also reproduced in a heterozygous combination of *IPO4[1]* with *Df(3L)ED223* deficiency that uncovers the *Apl* – *Arts* locus. *D*, a defective loading of Mst77F in spermatids associated with abnormal nuclear morphology and spermiogenesis arrest in mutant testes that do not express APL or ARTS. Confocal images of testes from *Df(3L)IPO4[2]/TM6B* heterozygote (control) and homozygous mutant *Df(3L)IPO4[1]* and *Df(3L)IPO4[2]* males stained with DAPI, Mst77F (31), and ProtB (6) antibodies. Mst77F staining is severely reduced in spermatid nuclei of the mutants, unlike the ProtB staining. In contrast to control seminal vesicles that contain mature, needle-like sperm, mutant seminal vesicles do not contain sperm, and the only visible nuclei are the somatic nuclei of the flattened vesicles. *hatched yellow boxes*, magnified view areas of spermatid staining; *white*, DAPI staining of DNA; *cyan*, Mst77F staining; *green*, ProtB staining; scale bars, 50 or 10 μm. *E*, defective loading of Mst77F in spermatids of the null mutant allele of *Apl* (16). The loading of mRFP1-Mst77F and ProtB-GFP transgenic proteins was detected by autofluorescence and confocal microscopy. *Blue*, DAPI staining of DNA; *red or white*, mRFP1-Mst77F; *green or white*, ProtB-GFP; scale bar, 10 μm. APL, APOLLO; ARTS, ARTEMIS; DAPI, 4′,6-diamidino-2-phenylindole; IF, indirect immunofluorescence; IPO4, importin-4; Mst77F, male-specific transcript 77F; NAP1, nucleosome assembly protein 1; SV, seminal vesicle; TAP/p32, HIV-1 Tat-associated protein/p32.

To analyze the roles that *Drosophila* IPO4 orthologs ARTS and APL may play in sperm chromatin metabolism and loading of Mst77F in sperm chromatin *in vivo*, we used CRISPR/Cas9 to prepare fly mutant alleles that inactivated both *Apl* and *Arts*. A guide RNA complementary to both genes (Fig. 4*A*) was expressed in the female germline together with the Cas9 enzyme (20). Three alleles that encompassed mutations in both *Apl* and *Arts* were recovered (Fig. 4*A*). *Df(3L)IPO4[1]* encompasses a deficiency completely deleting *Apl* (and flanking sequences) as well as a small deletion (indel) in *Arts*, resulting in a frameshift in its open reading frame. *Df(3L)IPO4[2]* encompasses a deficiency uncovering the central portion of the *Apl*–*Arts* locus; there is also an indel producing a frameshift in the putative *Apl–Arts* “fusion gene”. Additionally, *IPO4[3]* contains frame-shifting indels in both genes (Fig. 4*A*). Since the frameshifts eliminate the protein-binding KAP95 domains of both proteins, we expected these mutations to result in the loss of function for both genes.

We also raised a polyclonal antibody (IPO4-C, Fig. 4*A*) that recognizes both recombinant APL and ARTS (Fig. S4*A*). When lysates of whole adult males (strongly expressing APL in the testis) and adult females (strongly expressing ARTS in ovaries) were examined by western blot, the antibody specifically stained an ∼120-kDa band in wildtype animals but failed to detect APL or ARTS in *Df(3L)IPO4[1]* and *Df(3L)IPO4[2]* (Fig. S4*B*). Consistent with the putative function of APL in loading of Mst77F into sperm chromatin, IPO4-C antibody strongly stained elongating spermatid nuclei of wildtype males (Fig. 4*B*). In contrast, no nuclear staining was evident in mutant spermatids (Fig. 4*B*). Also, as expected, robust cytoplasmic staining of ARTS was observed in wildtype but not *Df(3L)IPO4[1]* or *Df(3L)IPO4[2]* ovaries (Fig. S4*C*).

Homozygous mutant males were completely sterile, producing no progeny in crosses with control females (Fig. 4*C*). In addition, no mature, needle-shaped sperm was observed in their seminal vesicles (Fig. 4*D*). Early spermatogenesis appeared normal in homozygous *Df(3L)IPO4[2]* males, but shortly after the histone-to-SNBP transition, spermatid nuclei all appeared abnormally shaped and condensed. The spermiogenic phenotype appeared much more severe in *Df(3L)IPO4[1]* mutants, with testes containing rare, scattered spermatid nuclei. Interestingly, in both mutants, while ProtB was nevertheless loaded in spermatid nuclei, Mst77F remained undetected by immunofluorescence (Fig. 4*D*). It has been previously reported that *Apl* null mutations do not affect the loading of Mst77F in spermatid chromatin (16). In contrast, by using an mRFP1-Mst77F transgene, we observed that Mst77F incorporation in *Apl[null2]* spermatids was, in fact, severely reduced (Fig. 4*E*). However, by intentionally increasing the intensity of red florescence detection on confocal images (10-fold over that used for control samples), mRFP1-Mst77F could be detected in mutant spermatids (Fig. S4*D*), indicative of a very low-level deposition of Mst77F. This result may explain the apparent discrepancy between the two studies.

Unexpectedly, whereas all three mutant alleles are male sterile, only *Df(3L)IPO4[1]* mutation impairs the female fertility (Fig. S4*E*), despite neither allele expressing the full-length ARTS (Fig. S4, *B* and *C*). The result seemingly contradicts the previous report that *Arts* is required for female fertility (16). However, the homozygous *IPO4* mutants can potentially express truncated versions of APL and/or ARTS that encompass IBN_N but not KAP95 domains of the protein (Fig. 4*A*). Thus, the truncated protein is likely sufficient to mediate the function(s) of ARTS in the egg. We also noted that the female sterile *Df(3L)IPO4[1]* fails to manifest the “round egg” phenotype observed for the progeny of homozygous *Arts[null1]* mothers (16) (*data not shown*). Combined, these observations indicate that the female sterility of *Df(3L)IPO4[1]* does not arise from the mutation of *Arts*. Despite a high homology between *spd-2* and *CR18217* nucleotide sequences flanking *Apl* near the distal breakpoint in *Df(3L)IPO4[1]*, *CR18217* transcript cannot be translated into a functional protein due to the presence of multiple stop codons. For instance, there is a small 31-bp deletion in the *CR18217* sequence, compared to that of *spd-2* (Fig. S4*F*). This deletion is retained in the *Df(3L)IPO4[1]* genome and predicted to bring about a premature termination of any hypothetical fusion protein and thus inactivation of *spd-2* (Fig. S4*F*). Notably, *spd*-*2*-null mutant has been shown to exhibit male and female sterility (21). Therefore, the mutation of *spd-2* may explain the sterility of *Df(3L)IPO4[1]* females (Fig. S4*E*) as well as the stronger spermiogenesis failure phenotype in the *Df(3L)IPO4[1]* testis (Fig. 4*D*).

## Discussion

Our work identifies the *Drosophila* testis-specific IPO4 ortholog APL as a chaperone that mediates the deposition of protamine-like Mst77F on DNA or chromatin. It is also required *in vivo* for loading of Mst77F into sperm chromatin in differentiating sperm cells. It was reported previously that the *Drosophila* testis-specific chaperone tNAP and chromatin remodeler ISWI are required for Mst77F loading in the nuclei of late-canoe stage spermatids (2). Unlike the mutation of *Apl* though, the RNAi-dependent depletion of tNAP or ISWI also dramatically reduced the incorporation of ProtA and ProtB (2). Unfortunately, biochemical evidence for the function of these factors in SNBP deposition was lacking, therefore not excluding an indirect effect. In contrast, we demonstrate in a defined system *in vitro* that APL can mediate both the assembly of DNA–Mst77F complexes and the disassembly of nucleosomes (Fig. 3).

Previously, human IPO4 was shown to co-IP from HeLa nuclear extracts with H3-H4 histone dimers containing both the canonical (H3.1) and replacement-type (H3.3) histones (22). Thus, it was proposed to be involved in the nuclear transport of histones or histone-containing complexes. Similarly, the yeast IPO4 (Kap123p) and importin-5 (Kap121p) have been implicated in the nuclear transport of H3-H4 histone tetramers (23). Additional members of the importin β/NTR family of proteins, such as Impβ, Kapβ2, importin-7, and importin-9/Kap114p, can bind and transport other core and linker histones (24). In HeLa cells, IPO4 can form a complex with the histone chaperone ASF1B and newly synthesized histones H3 and H4, suggesting its role in the delivery of ASF1B-histone complex to the nucleus (25). Unexpectedly, a knockdown of IPO4 does not cause an accumulation of cytosolic histones or ASF1B (25). Thus, IPO4 may be involved in other aspects of chromatin metabolism, in addition to its predicted role in the nuclear transport. Our observations indicate that *Drosophila* IPO4 APL expels histones from chromatin *in vitro* and mediates this reaction independently of known histone chaperones. Our work reveals that APL functions as a dual chaperone for Mst77F and core histone, exhibiting a higher affinity toward histones. Therefore, in *Drosophila* spermatids, it could directly mediate the histone-to-SNBP exchange, which takes place during sperm chromatin assembly and maturation.

We do not observe a gross defect in the removal of core histones from spermatid or mature sperm nuclei of *Apl* or *IPO4* mutants (*not shown*). This finding is consistent with Mst77F representing only a fraction of the SNBP composition of sperm chromatin, so that only a percentage of total histones need to be evicted to accommodate for the newly deposited Mst77F. The deposition of Mst77F in the late canoe stage of spermiogenesis largely coincides with that of the transition protein Tpl94D (26), which is consequently removed and replaced in mature sperm by ProtA and ProtB. Thus, the majority of core histones may be expelled from the spermatid chromatin by a histone-for-Tpl94D exchange, likely mediated by factor(s) other than APL.

Interestingly, the loss-of-function mutation of *Apl* in flies phenocopies amorphic mutations of *Mst77F* (6), without any other notable phenotypic abnormalities, indicating that the loading of Mst77F into sperm chromatin is the major, if not the only, biological function of APL. It has been noted previously that mutations of certain NTR genes in model organisms give rise to defects in a range of cell type–specific biological processes (12), which was interpreted as evidence for a restricted cargo specificity of particular importins β. For instance, mammalian importins 5 and 13 are uniquely required for spermatogenesis, owing to their interactions with and timely transport of essential sperm-specific cargo proteins (27). Also, in *Drosophila*, a null mutation of importin-9 ortholog *Ipo9*/*Ranbp9* results in female and male sterility due to chromosome segregation defects in meiosis and disruption of the nuclear localization of several proteasome components thought to be involved in histone removal during spermiogenesis (28). Our data moreover suggest that in addition to the cargo transport, NTRs can execute auxiliary, specialized biochemical programs. For example, *Drosophila* APL can directly mediate the loading of Mst77F on DNA and the simultaneous release of core histones from chromatin. Importantly, since our defined system does not contain the Ran GTPase, this cargo exchange reaction can be carried out in a RanGTP-independent fashion but driven by the biochemical properties of the NTR alone.

ARTS, the ovary-specific paralog of APL, similarly facilitates the deposition of Mst77F on DNA *in vitro* (Fig. 3*B*), predictably so, considering the near structural identity of the two proteins. However, it is highly unlikely that this biochemical activity of ARTS has physiological consequences for chromatin structure in the *Drosophila* egg, where it is abundantly present. First, in steady-state Mst77F binding experiments with embryonic extracts (Fig. 1*D*), Mst77F-dissociating chaperones NAP1 and TAP/p32 appear to be in >5-fold molar excess compared to the Mst77F-loading ARTS. The reaction equilibrium (Figs. 2*D* and 3*E*) is further shifted toward the removal of Mst77F by a much greater concentration of core histones (maternally loaded) than that of Mst77F (contributed by a single sperm cell in the fertilized egg). Thus, rather than the disassembly of nucleosomes and deposition of Mst77F, ARTS may be required in the embryo to play other roles, such as the regulation or nuclear transport of essential components of actin networks, as proposed (16).

## Experimental procedures

### Recombinant proteins and antibodies

A bacterial expression construct for purification of V5-tagged Mst77F was prepared as described for ProtA/B (18). Baculovirus constructs for purification of FLAG-ARTS and FLAG-APL were prepared by PCR and molecular cloning. Rabbit polyclonal antibody that recognizes the C terminus of both ARTS and APL (IPO4-C) was raised by immunization with a conserved C-terminal polypeptide fragment of ARTS. See Supplementary Experimental Procedures for details of cloning, expression in *E. coli* or Sf9 cells and purification.

### Purification of Mst77F chaperones

DNA-Mst77F substrate (see Supplementary Experimental Procedures) equivalent to 10 μg of plasmid DNA (5 μg Mst77F-V5) was treated with 4 ml S-190 extract (29) (∼40 mg total protein) in a buffer containing 3 mM ATP, 30 mM phosphocreatine, and 2 μg creatine phosphokinase as described previously (29). The reaction products were fractionated by sucrose gradient sedimentation. 40-ml linear gradients of 5 to 45% sucrose in 25 mM Hepes-K^+^, pH 7.6, 0.1 mM EDTA, 150 mM NaCl, 1 mM DTT, 0.01% NP-40 0.2 mM phenylmethylsulfonyl fluoride, and 0.5 mM benzamidine were prepared and centrifuged for 20 h at 28,000 rpm (141,000*g*) in SW-28 rotor (Beckman) at 4 °C. As controls, S-190 extract alone or DNA-Mst77F mixed with 20 mg nuclease-free BSA (New England Biolabs) were similarly analyzed by sucrose gradient sedimentation. The gradients were cut in 3-ml fractions, and the fractions (2-μl aliquots) were analyzed by anti-V5 western blotting. Mouse monoclonal V5 antibody (Sigma) and infrared dye-labeled secondary antibody (LI-COR Bioscience) were used at 1:5000 and 1:10,000, respectively.

For purification of putative Mst77F chaperones, gradient fractions from the top of the gradients that contained V5-immunoreactive material were pooled and immunoprecipitated with 20 μl anti-V5 agarose (Sigma) as described (18). Similar fractions of control, S-190-only, sucrose gradients were also pulled down. The immunoprecipitated material was eluted with 40 μl of 0.2 M glycine, pH 2.0, neutralized with 5 μl 1 M Tris-base and analyzed by SDS-PAGE (4–20% gradient polyacrylamide gel, 20 μl eluate per lane) and Coomassie staining. Protein identities in prominent bands were determined by mass spectrometry.

### Protein–protein interactions, remodeling of DNA-Mst77F substrate, and Mst77F loading on DNA *in vitro*

Physical interactions between Mst77F and chaperones were analyzed by co-IP and glycerol gradient cosedimentation of recombinant proteins. The association of Mst77F with and its release from DNA was examined by size-exclusion chromatography and western with anti-V5 antibody as described (18). See Supplementary Experimental Procedures for details.

### IF staining of *Drosophila* testes and ovaries

*Drosophila* testes and ovaries were stained with IPO4-C, anti-Mst77F, anti-Mst35B, or anti-histone antibodies exactly as described (6, 30). See Supplementary Experimental Procedures for details.

## Data availability

All data are included in the manuscript.

## Supporting information

This article contains supporting information (32, 33, 34).

## Conflict of interest

The authors declare that they have no conflicts of interest with the contents of this article.
