# Supplementary material for: APOLLO, a testis-specific Drosophila ortholog of importin-4, mediates the loading of protamine-like protein Mst77F into sperm chromatin
Source: J Biol Chem. 2023 Sep 2;299(10):105212. doi: 10.1016/j.jbc.2023.105212 (PMC10520872; doi:10.1016/j.jbc.2023.105212)
Supplement: Supporting Information [file mmc1.pdf]

Supporting Material

APOLLO, a testis-specific *Drosophila* ortholog of Importin-4, mediates the loading of protamine-like protein Mst77F into sperm chromatin

Alexander V. Emelyanov, Daniel Barcenilla-Merino, Benjamin Loppin, and Dmitry V. Fyodorov

Supplementary Experimental Procedures

Recombinant proteins

Full-length open reading frame (ORF) of *Mst77F* was amplified from Mst77F-RA cDNA RH09844 (*Drosophila* Genomics Resource Center, DGRC) using Q5 polymerase (New England Biolabs) and the following PCR primers with NdeI and XhoI restriction sites on the 5'- and 3'-ends, respectively (underlined): N-Mst77F+ TCcatATGAGTAATCTGAAACAAAAGG and X-Mst77F- AActcgagCATCGAGCACTTGGGCTTGG. The coding sequence was cloned in a T7-driven expression construct in frame with the C-terminal V5 tag, intein and chitin binding domain as described (18). Mst77F-V5 was expressed in *E. coli* (Rosetta 2) and isolated by successive steps of chitin affinity chromatography, intein self-cleavage and cation exchange (Source 15S) FPLC (18). The sequence of purified, intein-processed protein is as follows: Mst77F-PA(1..215)-L**EGKPIPNPLLGLDST**GASVDG. V5 tag sequence is shown in bold.

To express FLAG-tagged ARTS, its full-length ORF was modified to include an N-terminal NdeI site by cloning a 248-bp gBlock (IDT, Inc., EcoRI, NdeI and StuI sites are underlined): CTCTTTTTCGCGAATTCCTCCCCGGTATCCCACTTTTCAATTCTCAATTCCGCGTAACGTCG GCTGTAATTTGTACCGGATTGCATTGGATTATCCGCCTGGGCGCGAACAGTAACTCCCCAc AtATGGAGGCAGCTATTCTGGATATAATCAACGGGATCCTGGCCATAGACACGGAACGAAT CCGTGAGTCCACAGCCAAGATGCTAAAGGCCTACGAGAATCCCGATTCTCTGCTGGTTCTC ACC – into *Arts-RB* cDNA plasmid RE37107 (DGRC) digested with EcoRI and StuI. This construct was digested with NdeI (partially) and HindIII. A 3,333-bp NdeI-HindIII fragment was cloned into pFastBac vector (Thermo Fisher) in frame with the N-terminal FLAG tag as described (32). *Apl-RA* cDNA was synthesized as two 1,638-bp gBlocks (IDT, Inc.): GACAAGCATATGGAGGCAGCTATTCTGGATATAATCAACGGGATCCTGACCATAGACACGG AACGAATCCGTGAGTCCACAGCCAAGATGCTAAAGGCCTACGAGAATCCCGATTCTCTGCT GGTTCTCACCCAGATCGTGATGTCGGACAGGCCGGTCCAAGAGCGGCAGGTCGCCGCCGT GCTCCTCAAGAGGCGGGTCAAAAAGTTACGCCACTGGCAGTTGGTTCCCGCCGAGCACCA GGCAGCAATCAAATCGAACATGCTACAGGTCCTCATCGCGGTGAAGGAAAAGACCGTGAAG

GGCACGGTGGCCTTTATAATTGGATCGCTGGTGCGCCACGAGGAGGGCGAGCAAAACTCC
TGGAGGGAGGAAATACTGAAGTTTATTTACGAACGTTGCAGCAGCCCCGATCCAATAGAAA
GCGAGCGGGGCAGCTCAATCTTTTCCTCGCTCATGGACGCTGCTCCCGATCAGTTTTCGGA
CCACACGGATACTATGTTCCCATTGCTTGCCGGCATTCTAGTTACCGCCGAGGCGAATGGG
AACATGGCCACACCCACCGTGCATAACATGCTGACCGGTTCATGCTTCTTGTTGCCTTTCGT
AAGTGGACATAGTAATGCTGAGCAGATTGTGGTTAAGGCTGTGCCGCTCATACTCAAGGCC
CTGGCCGCCTTTGTCGAAAAGGGGTACAGTATTGAGTTCATGGGTGCCTTTGATATCATCGA
CAGCATGGCCGAGCATGTGCCCCACTTGCTGACCGGTAATGTGAAGCTGCTTCTGGAGTTT
TGTCTGATGATTGCGAGAAACAAGCAGTTCGACGCTTCGATTCGGGTTCAGGTGCTTACCTT
TGTAGGCAGCCTAGTTCGCCTCAAGAAGAAAATTATTATGAAGCAGAAACTACTACAGCCCA
CACTATCTGTTCTTTTCGAAGTGATTTGCCAGGACGACCTCGAGGAAGGTGACGATGATTAC
TTTTCCTCGGAGAGCCTGAGTAGTCCATCCAATGCAGCTGCGCAGACACTGGACCTGATGG
CCCTTCACATGGTGCCGGACAAGTTTATTCCGCCACTACTGGATTTGCTGGAGCCTGCGTT
GCAGAGCCCGGAACCTGTGCTTCGCCGTTCCTCTTTTATTTGCATGGGCGTTATTGCCGAG
GGCTGTTCCGAGGCCATTGGGAAAAAGTATCTCGAGGTCATGCTAAATATCATCAAAGCTG
GAGTGTTGGACTCTGTTATGTTCGTGCGGACCGCCGCATTCTTCGCCCTCGGTCAGTTTTC
CGAGTTTCTTCAGCCGACGATCTGCAAGTTTGCTCCCCAAATTCTACCCGTGTTGTTCGACT
ATCTAAACCAGCTGGTACTGGAACTCAAGGTTGGTGAGCCGGATTCGAAACATATGGATCG
TATGTTTTATGCCCTGGAGACCTTTTGCGAGAATCTGGATGAGGATATTGTTCCTTATTTACC
CACACTTATGGATCGTCTGTTTGGGGTTATGGAACCGCAGAACTCAAATCAAATGCGTGAAA
TGGCTTTGTCGGCCATAGCAGCGGTGTCAGCAGCAGCTAAGGAAAACCTGATGCCATATTT
CCCCAGGATCATGACCGTACTACAGGGCTGTCTGGTGAAGGATTGC

and

GCTGTCTGGTGAAGGATTGCCCAAAAGAAATGTACAGTCAGCGCATCCAAGCTATCGACAC
TCTGGCCGCGCTGTGTCGCGAATTGGGCAAGGATAATATTATTCCTCTTGCCGACGATACA
ATGAACTTCTGCCTGATGATGCTGGAAGACGGTCCGGATGATCCCGAGTACCGCAGGAGTA
TATACAACCTGATGTCCTCCCTGTCATCAGTTGTCAACGAGAGCATGGCCAGTGTGTTCCCC

AAGTTCATTGATCGCATAATGGAGTCTGTGATTTTTTCGGAGGACATGGTGCCAAATGTGTC
GGACAATGCGGACGATGATTTGGCCCTAGTAGATGCCCCCGACATAGAGATTGATTTAGAG
CACACAGACGACGAAGATGACCAAGACGCTTACCCGGTGGAGAATGATTATATCGTCGAAA
AGGAGGAGGCCATTCTGTCACTCAAGGAGTTTGCTACCCATACCGGCGCCGCCTTCGCGC
CCTATTTGCAATCTGCATTCGAGAACGTCTACAAGATGATTGATCATCCGCAAGGCGACGTT
CGTATGGCATGTATTGACTCCATTTGCTCGTTCATTACGGCTCTCCACAAGTTGGATGATGC
CGCCGGCCTGAAGCGCGCCTGCGAGATTGCCATTCCAAAGTTTGCACATATAATGCGCACC
GACGACCAGGTTGCGGTCGTACTCCGCATGCTTGACGTCCTCTACGATGTCTTCAAGTACG
TGCCGGCGATAAACAGCCAGGAGCACGCCGAACTTATATTCGGTTGCATCAGGGACATCTT
CACAAATAAAATGGCCTGTCAGTTTAACGAGGAGAGCGGCGGCGGAGATGATGAGTGTTCG
GAGGAAAGCGAGAACGACGAGATGCTGTTCGAGAACGCTGCCAATCTGTTCCCCATGTTTG
GCTTAACCCTTCAGCCGGAGCTGTTCTCGCTTTATTTTGGACGCCTTTACCATTTCTATATTC
AAAGGCTGGCGAAGGTAAAGGAGCGCGATTTACCAGAACAGCGGGCATACATCTATGGCG
CACTTGCTGACTGCTGCAAGGCGTTAAAAGGTTGCTGTGCTACTTATTTCGATGCCCTACGT
CCCATTTTTATCGCCGGTTCCAGGGATTCTGATGCCAAAGCGCGACAGAACTCCTATTTTGC
ACTAGGCGAAATTGTCTTCCATTCCGAGGAAAAATCATTCGAGTCTTATCCGACAATTTTGCA
AGCCCTTTCCGAAGCAATTGTCAGGGAATCCGTTCCTGCCGCCATGGACAACATTTGCGGA
GCAGTTGCTCGTCTCATAGTTACCAACCCAGATTCAGTGCCGCTTGGCCAGGTGCTGCCCG
TGTGGCTCAACCATTTGCCACTGAAGGATGACACCGTGGAGAACGATGTGATTCAGAAGGC
GTTCCGTGTGCTATACTTGAAGGCCCGCCCCAGCATTGAGGCACATCTTGAGCAAATCCTG
GCCATCACCATAGAGGCGAGTTACAAGAGACAGATGCCTGACGTCGAGACAACCGAGAGC
GCGGTGGCCCTCATCAAAGAGATTCGCGCCAACTACCCGGAACTGTTCAGCAAAGTATCGA
ACATGAATCCAGAAGTGTTCAATTATGTGCAAGCCCTGTAAGCCC (translation start and stop codons are underlined). The complete ORF was cloned in pFastBac vector downstream of the FLAG tag in place of *Arts-RB* by Gibson assembly. The expression constructs encode the following polypeptides: M**DYKDDDDK**H-ARTS-PB(1..1,080) and M**DYKDDDDK**H-APL-PA(1..1,080). FLAG tag sequences are shown in bold.

Recombinant baculoviruses were produced by standard methods (Thermo Fisher), amplified and used to infect Sf9 cells in suspension culture (Sf-900 II SFM medium, Gibco), 10^6 cells/ml. Cells were harvested 72 h post-infection, and the proteins were purified by successive steps of FLAG affinity chromatography and anion exchange (Source 15Q) FPLC as described (33). See (18) for the *E. coli* expression and purification protocol of NAP1-His_6, TAP/p32-His_6 and NLP-His_6.

Proteins were analyzed by SDS-PAGE on 4-20% gradient polyacrylamide (PAA) gels and Coomassie staining along with a range of BSA mass standard (Pierce). Protein concentrations were determined by infrared scanning (Odyssey Fc Imaging System, LI-COR Biosciences) and interpolation relative to the mass standard. All proteins were aliquoted, flash-frozen in liquid nitrogen and stored at -80°C in their final ion exchange chromatography fractionation buffers: HEG (25 mM HEPES-K^+, pH 7.6, 0.1 mM EDTA, 10% glycerol) additionally containing 0.02% NP-40, 1 mM DTT, 0.2 mM PMSF, 0.5 mM benzamidine and 300-500 mM NaCl.

IPO4-C antibody

IPO4-C fragment encompassing amino acid residues 932-1,080 of ARTS-PB, N-terminally tagged with five histidine residues was cloned from a 494-bp gBlock (IDT, Inc.):

ccctcatATGcaccatcatcatcatCACTCAGAAGAGAAGTCATTCGAGAGCTATCCAACAATCCTGCA
AGCTTTATCCGAGGCCATTGTACGTGAGAGTGTCCCGGCAGCCATGGATAACATTTGTGG
CGCGGTCGCGCGTTTAATTGTGACGAACCCCGACAGCGTGCCTTTAGGCCAGGTACTTCC
TGTATGGTTAAACCATTTACCGCTGAAAGATGACACAGTAGAGAATGATGTTATTCAGAAGG
CTTTCCGTGTACTTTACCTGAAAGCTCGTCCCTCGATTGAGGCGCATCTGGAGCAGATTCT
GGCTATTACTATTGAAGCGAGCTACAAAAAGCAAATGCCCGATGTTGAGACTACTGAATCG
GCTGTTGCGTTGATTAAAGAAATCCGCGCGAATTACCCCGAGCTGTTTAGCAAGGTATCAA
ACATGAATCCGGAGGTTTTCAACTACGTCCAAGCGCTGTGATAActcgagtcctcctcct

(translation start and stop codons as well as NdeI and XhoI restriction sites are underlined). NdeI-XhoI digestion fragment was ligated into pET-24b construct. The ~17.5-kDa polypeptide:

M**HHHHH**HSEEKSFESYPTILQALSEAIVRESVPAAMDNICGAVARLIVTNPDSVPLGQVLPVWL NHLPLKDDTVENDVIQKAFRVLYLKARPSIEAHLEQILAITIEASYKKQMPDVETTESAVALIKEIR ANYPELFSKVSNMNPEVFNYVQAL, M**HHHHH**-ARTS-PB(932..1,080) – contains a single mismatch with the corresponding APL-PA sequence (ARTS-PB-K1037 versus APL-PA-R1037). The IPO4-C fragment was expressed in *E. coli* as described above, purified in denaturing conditions (in 6 M urea) on Ni-NTA resin (Qiagen), dialyzed into PBS and used as an antigen to raise rabbit polyclonal antibodies. Animal injections and serum collections were performed by Pocono Rabbit Farm & Lab.

For western blot analyses of recombinant proteins (**Suppl. Fig. S4A**), ~20 ng purified recombinant FLAG-APL and FLAG-ARTS each were resolved on an SDS-PAGE gel (10% PAA) and probed with the IPO4-C antibody (1:5,000) and infrared dye-labeled secondary antibody (LI-COR Bioscience, 1:10,000). Images were obtained using the LI-COR Odyssey Infrared Imaging System.

Reconstitution of DNA-Mst77F substrate

The substrates were reconstituted as described (18). Supercoiled pGIE-0 plasmid DNA (~3.2 kbp) was mixed with NaCl (to 2 M) and combined with Mst77F-V5 in a 2 M NaCl-containing buffer. The mass ratio of Mst77F to DNA was ~0.5:1, empirically sufficient to neutralize ~50% of the DNA negative charge. The mixtures additionally contained nuclease-free BSA (NEB) at 0.1 mg/ml. They were dialyzed overnight at 4°C into HEG with 150 mM NaCl, 0.02% NaN_3, 0.02% NP-40, 1 mM DTT, 0.2 mM PMSF and 0.5 mM benzamidine. Some control dialysis mixtures contained only DNA but no Mst77F. The substrates were stored at 4°C for up to four weeks.

DNA-Mst77F substrate remodeling with recombinant factors in vitro

Remodeling was assayed *in vitro* as described previously (18) in 200-µl Reaction Buffer (HEG + 150 mM NaCl, 0.02% NP-40, 1 mM DTT, 0.2 mM PMSF, 0.5 mM benzamidine) that

contained 0.1 mg/ml BSA, 0.5 pmol DNA-Mst77F substrate (~1 µg DNA, equivalent to 0.1 µM Mst77F-V5), 0 or 0.4 µM FLAG-ARTS, 0, 0.2 or 1 µM NAP1-His_6, 0 or 1 µM TAP/p32-His_6 and/or 0 or 1 µM NLP-His_6. The reactions were incubated at 27°C for 2 h and applied to 2-ml Sephacryl S-500 gravity flow gel filtration gravity-flow drip columns equilibrated to Reaction Buffer (**Fig. 2A**). After discarding the void volume (0.5 ml), eight 200-µl chromatographic fractions were collected, and 20 µl were analyzed by SDS-PAGE (15% PAA) and western blotting with mouse monoclonal V5 antibody (Sigma, 1:5,000) and infrared dye-labeled secondary antibody (LI-COR Bioscience, 1:10,000). Images were obtained using the LI-COR Odyssey Infrared Imaging System.

Mst77F loading on DNA in vitro

Drosophila native core histones were purified from 0-12 h embryos and quantified as described (29). Oligonucleosome substrate (salt-dialyzed chromatin) was prepared from native core histones and pGIE-0 plasmid DNA by salt dialysis as described (34).

Mst77F loading was assayed *in vitro* in 200-µl Reaction Buffer (HEG + 150 mM NaCl, 0.02% NP-40, 1 mM DTT, 0.2 mM PMSF, 0.5 mM benzamidine) that contained 1 pmol DNA or salt-dialyzed chromatin (~2 µg DNA, 0 or ~2 µg total core histones), 0.1 mg/ml BSA, 0.1 µM Mst77F-V5, 0.4 µM FLAG-ARTS, 0.4 µM FLAG-APL, 1 µM NAP1-His_6, 1 µM TAP/p32-His_6 or 1 µM NLP-His_6. Mst77F-V5 was incubated with chaperones for 30 min at 4°C, and DNA/chromatin was mixed into reactions. The reactions were incubated at 27°C for additional 2 h and applied to 2-ml Sephacryl S-500 columns (**Fig. 3A**). After discarding the void volume (0.5 ml), eight 200-µl chromatographic fractions were collected, and 20 µl were analyzed by SDS-PAGE (15% PAA) and western blotting with mouse monoclonal V5 antibody (Sigma, 1:5,000) and rabbit polyclonal FLAG antibody (Sigma, 1:1,000) followed by corresponding infrared dye-labeled secondary antibodies (LI-COR Bioscience, 1:10,000). Images were obtained using the LI-COR Odyssey Infrared Imaging System. To analyze the fractionation of plasmid DNA (as the DNA-Mst77F complex), 50-µl aliquots of column fractions were treated with proteinase K, extracted with

phenol-chloroform, precipitated with ethanol and examined on an ethidium-stained agarose gel (**Suppl. Fig. S3B**).

To assay for core histone removal from the chromatin substrate, reactions were performed and fractionated as above with or without 0.4 μM FLAG-APL and 0.1 μM Mst77F-V5. 200-μl Sephacryl column fractions were precipitated with acetone and analyzed by SDS-PAGE and Coomassie staining.

Protein-protein interaction analyses

For Mst77F-chaperone co-IP analyses, 0 or 1.25 μg Mst77F-V5 (0.4 μM) was incubated with 25 μg FLAG-ARTS (1 μM), 22 μg NAP1-His_6 (2.5 μM), 15 μg TAP/p32-His_6 (2.5 μM) or 9 μg NLP-His_6 (2.5 μM) in 200-μl Reaction Buffer (HEG + 150 mM NaCl, 0.02% NP-40, 1 mM DTT, 0.2 mM PMSF, 0.5 mM benzamidine) in the presence of 0.2 mg/ml BSA for 1 h at 4°C. 20 μl V5-agarose resin (Sigma) was added, and the mixtures were incubated for additional 1 h at 4°C. The resin was washed three times with 400 μl Reaction Buffer (without BSA), and the proteins were eluted with 30 μl 0.2 M glycine. The eluates were neutralized with 6 μl 1.5 M Tris-HCl, pH 8.8, mixed with 12 μl 4x SDS-PAGE loading buffer, boiled, and one-third (16 μl) of each sample was resolved on SDS-PAGE (4-20% gradient) gel and stained with Coomassie.

For glycerol gradient sedimentation assays, 10 μg (1 μM) Mst77F-V5 and/or 22 μg core histones (0.5 μM tetramers, 1 μM dimers) were incubated with 0 or 20 μg (0.4 μM) FLAG-APL for 30 min at 4°C in 400 μl Glycerol Gradient (GG) buffer (25 mM HEPES-K^+, pH 7.6, 0.1 mM EDTA, 500 mM NaCl, 0.02% NP-40, 1 mM DTT, 0.2 mM PMSF, 0.5 mM benzamidine) and loaded on ~5 ml linear gradients (10-40% glycerol in GG buffer). The gradients were ultracentrifuged for 16 h at 40,000 rpm (194,000 *g*) in SW-55 Ti rotor (Beckman) and cut into ten 0.5-ml fractions. 100 μl each fraction was precipitated with acetone and analyzed by SDS-PAGE (4-20% gradient PAA) and Coomassie staining.

Fly genetics and PCR analyses

Flies were grown on standard corn meal, sugar, and yeast medium with Tegosept. Stocks and crosses were maintained at 25°C. Fly stocks were obtained from M. Long (University of Chicago), NIG-FLY (Japan), Bloomington (IN, USA) and Kyoto (Japan) stock centers.

Apl and *Arts* mutations were generated by CRISPR/Cas9 exactly as described (20). Double-stranded oligonucleotide encompassing the guide RNA was produced by annealing gtcgCATGCTGTCGATGATATCAA with aaacTTGATATCATCGACAGCATG (lower case letters designate sticky ends) and ligated into BbsI-digested pCFD3 (20). The construct was injected in attP40 stock embryos by BestGene, Inc. *y v/Y; attP40{gRNA* v^+*}*/+ males were crossed with *y w; Sco/CyO* females, and *y w; attP40{gRNA* v^+*}/CyO* lines were established in two generations. Similarly, *y cho v/Y; Sp/CyO P{nos-Cas9* y^+ v^+*}2A* males (NIG-FLY/FlyCas9 CAS-0004) were used to generate *y w; Sco/CyO P{nos-Cas9* y^+ v^+*}2A* lines. These flies were crossed *inter se*, and then *y w/y w; attP40{gRNA* v^+*}/CyO P{nos-Cas9* y^+ v^+*}2A* females were crossed to *y w/Y; +/+; TM3 Sb/TM6B Tb* males. 40 individual *y w/Y; +/ CyO P{nos-Cas9* y^+ v^+*}2A; Mut?/TM3 Sb* males were crossed to *w/w; +/+; Df(3L)ED223/TM6C cu Sb* females, and fertility of *Mut?/Df(3L)ED223* males was tested in crosses with *y w/y w; +/+; TM3 Sb/TM6B Tb* females. *Df(3L)ED223* uncovers both *Apl* and *Arts*. 11 alleles that exhibited male sterility were genotyped by PCR and sequencing, and three alleles that encompassed mutations affecting both *Apl* and *Arts* were recovered (**Fig. 4A**). They were saved as *y w; +/+; Df(3L)IPO4[1]/TM6B Tb*, *y w; +/+; Df(3L)IPO4[2]/TM6B Tb* and *IPO4[3]/TM6B Tb* lines for further analyses.

The following primers were used for amplification of genomic DNA from *y w* control and homozygous mutant males: F1, GCCTCGGCTTCTGTCTCTAC (3L:16,593,322..16,593,341); F2, gctcgaatggacgcacac (3L:16,595,971..16,595,988, lower-case letters designate intronic sequences); F3, ttttccaagaatctttggtctctcatgttc (3L:16,603,651..16,603,680); R1, GGACTAACCGGACGTGGTTC (3L:16,608,817..16,608,798); R2, TCGAATCCGGCTCACCAAC (3L:16,598,045..16,598,027 and 3L:16,605,732..16,605,714); and R3, GTTGATTATATCCAGAATAGCTGCCTCC (3L:16,595,536..16,595,509 and 3L:16,603,214..16,603,187) – in all pairwise combinations. The PCR products were purified and

sequenced with F1; S2, CTGCTCGCGCAGCTTAC (3L:16,594,327..16,594,311 and 3L:16,601,996..16,601,980); S3, GCGGGTCAAAAAGTTAC (3L:16,596,206..16,596,222 and 3L:16,603,893.. 16,603,909); and/or S4, AAAGGTAAGCACCTGAAC (3L:16,596,908..16,596,891 and 16,604,595..16,604,577). The ability to amplify intervening sequences as well as sequencing information was used to determine the nature of lesions introduced by CRISPR/Cas9 (**Fig. 4A**).

Df(3L)IPO4[1] allele is predicted to express truncated proteins ARTS-PB(1..237)esstawpsmcptc* and MDrSSGSQrNGSyMDQNSLGILNvDNLKslrrlvqlq*, homologous to SPD-2(1..28)slrrlvqlq*. *Df(3L)IPO4[2]* allele is predicted to express truncated APL-PA(1..238)hrqhgrvcapladrqceaasgvlsddcekqavrrfdsgsgaylcgqpsspqeenyyeaetttahticsfrsdlpgrpqgr*. *IPO4[3]* allele is predicted to express truncated proteins APL-PA(1..237)esstawpsmcptc* and ARTS-PB(1..237)esstawpsmcptc*.

For western analyses (**Suppl. Fig. S4B**), *y w* control and homozygous *Df(3L)IPO4[1]* and *Df(3L)IPO4[2]* adult flies were lysed in SDS-PAGE loading buffer, and material equivalent to one adult male or female per lane was loaded on SDS-PAGE gels (10% PAA) and probed with the rabbit IPO4-C antibody (1:5,000), mouse anti-beta tubulin antibody (Developmental Studies Hybridoma Bank E7, 1:5,000 or 1:20,000) and corresponding infrared dye-labeled secondary antibodies (LI-COR Bioscience, 1:10,000). Images were obtained using the LI-COR Odyssey Infrared Imaging System.

For quantitative male and female fertility analyses, 10 single 0–1-day-old males (**Fig. 4C**) or virgin females (**Suppl. Fig. S4E**) were crossed with 10 *w[1118]* counterparts each. After two days, parents were discarded, and the total numbers of eggs laid by the mothers were counted in each vial. The numbers of hatched larval progenies were determined after 48-h incubation at 25°C.

Staining of Drosophila *testes and ovaries*

Testes and ovaries were dissected in PBS-T (1X PBS, 0.15% Triton) and fixed for 20 min in 4% formaldehyde at room temperature (RT). Tissues were washed three times in PBS-T and incubated with primary antibody overnight at 4°C. After three 20-min washes in PBS-T, they were incubated with secondary antibodies for 2 h at RT. They were then washed three times and mounted in mounting medium (DAKO) containing 1 µg/mL DAPI.

Primary antibodies were rabbit polyclonal anti-Mst77F [171–184] (1:1,000) (31), rabbit polyclonal anti-Mst35Bb (ProtB) (1:200) (6), rabbit polyclonal IPO4-C antiserum (1:1,000) and mouse monoclonal anti-histone (Millipore MABE71, 1:1,000). Secondary antibodies were goat anti-rabbit or mouse IgG (H + L), DyLight 488 conjugated (Thermo Scientific, 1:1,000).

Indirect immunofluorescence (IF) and GFP/RFP auto-fluorescence images were obtained using a LSM 800 confocal microscope (Zeiss). Images were treated with Zen imaging software (Zeiss), Image J or Photoshop CS2 (Adobe).

Supplementary References

32. Andreyeva, E. N., Emelyanov, A. V., Nevil, M., Sun, L., Vershilova, E., Hill, C. A. *et al.* (2022) *Drosophila* SUMM4 complex couples insulator function and DNA replication control eLife **11**, e81828 10.7554/eLife.81828

33. Emelyanov, A. V., and Fyodorov, D. V. (2016) Thioredoxin-dependent disulfide bond reduction is required for protamine eviction from sperm chromatin Genes Dev **30**, 2651-2656 10.1101/gad.290916.116

34. Lu, X., Wontakal, S. N., Kavi, H., Kim, B. J., Guzzardo, P. M., Emelyanov, A. V. *et al.* (2013) *Drosophila* H1 regulates the genetic activity of heterochromatin by recruitment of Su(var)3-9 Science **340**, 78-81 10.1126/science.1234654

A

Gel band(s)	FlyBase ID	Protein	Expected MW, kDa	Peptides	Unique peptides	Sequence coverage	MASCOT score
p122	FBgn0042177	**ARTS**	121	69	7	57%	16,224
p55	FBgn0015268	**NAP1**	43	32	32	76%	10,757
p30, p29	FBgn0086915	**Mst77F**	25	13	13	47%	1,087
p26	FBgn0034259	**TAP/p32**	29	7	7	41%	704
p22	FBgn0016685	**NLP**	17	10	10	53%	1,168

B

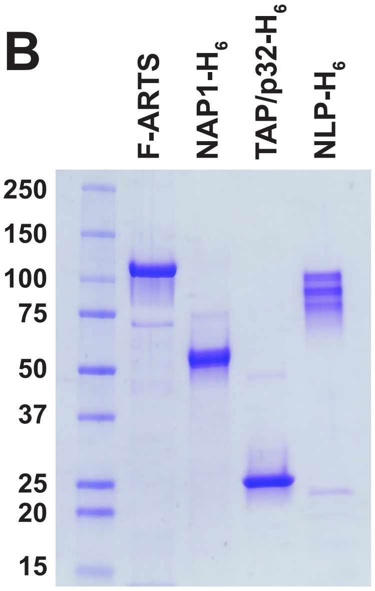

SDS-PAGE, Coomassie

C

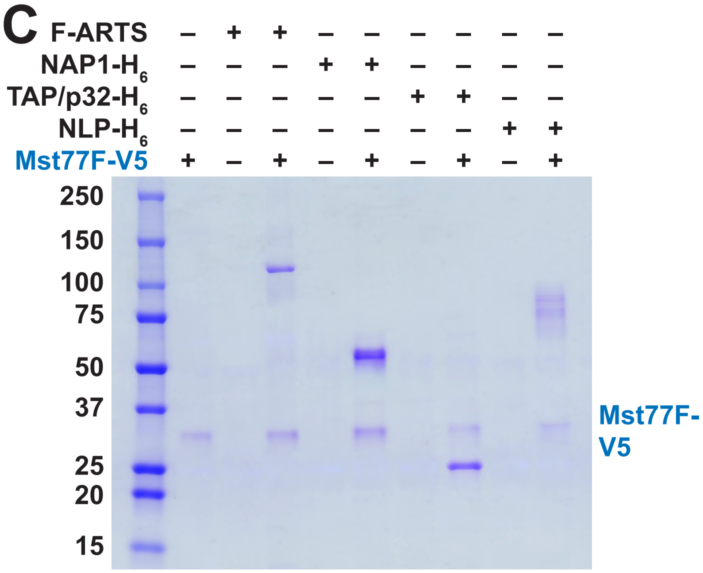

V5 pulldowns, SDS-PAGE, Coomassie

Suppl. Fig. S1. Mst77F eviction activity and purification of putative Mst77F chaperones from *Drosophila* embryonic extract.

(**A**) Protein identities and (poly)peptide statistics from mass spectroscopic analyses of protein bands from **Fig. 1D**.

(**B**) SDS-PAGE of purified recombinant Mst77F chaperones. ~1 µg each protein was loaded next to the molecular mass marker. Sizes (kDa) are shown on the left.

(**C**) Physical interactions of putative Mst77F chaperones with Mst77F-V5 analyzed by co-immunoprecipitation with V5 and SDS-PAGE. A molar excess of each putative chaperone was pulled down with V5 resin in the presence or absence of Mst77F-V5.

A

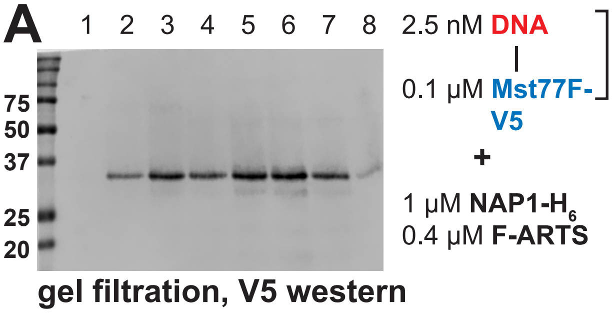

B

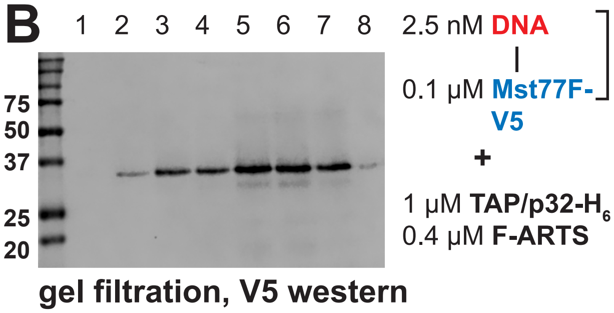

Suppl. Fig. S2. Remodeling of DNA-Mst77F-V5 substrate by putative Mst77F chaperones *in vitro*.

(**A**) ARTS inhibits NAP1-mediated Mst77F-V5 eviction from its complex with DNA. The experiment was performed and presented an in **Fig. 2B**. Compare to the NAP1 panel.

(**B**) ARTS inhibits TAP/p32-mediated Mst77F-V5 eviction from its complex with DNA. Compare to the TAP/p32 panel in **Fig. 2B**.

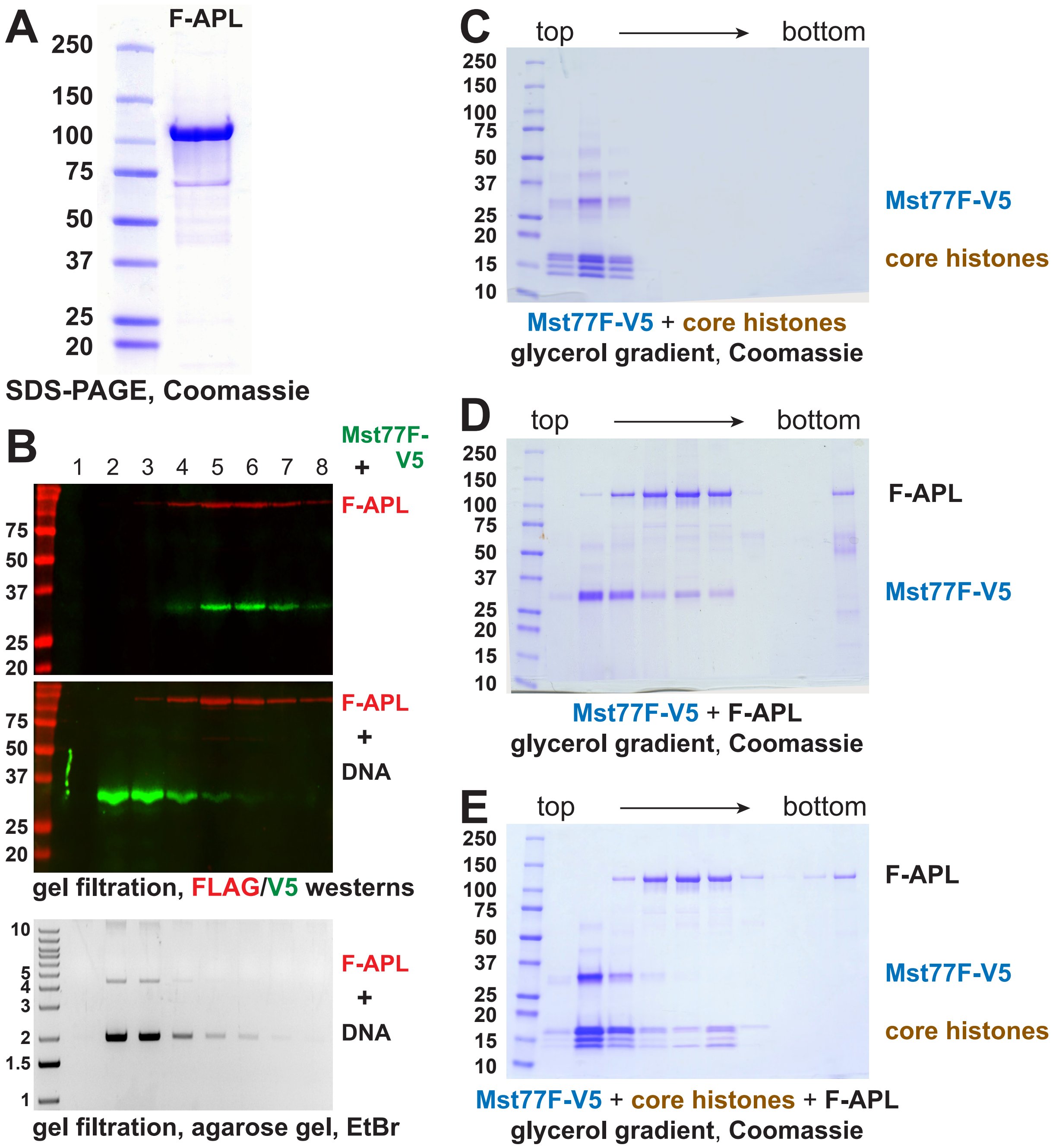

Suppl. Fig. S3. APL-mediated deposition of Mst77F-V5 on DNA and chromatin *in vitro*.
(**A**) SDS-PAGE of purified recombinant APL. ~1 µg protein was loaded next to the molecular mass marker. Sizes (kDa) are shown on the left.
(**B**) Size exclusion fractionation profiles of FLAG-APL, Mst77F-V5 and plasmid DNA before and after APL-mediated loading of Mst77F on DNA. Recombinant proteins in the fractions were examined by FLAG and V5 western blot. Plasmid DNA was detected by electrophoresis and ethidium staining. Red, FLAG western signal and protein molecular mass marker; green, V5 western signal. Compare directly to **Fig. 3B**, top two panels, which present the V5 western results (green channel). DNA marker sizes are indicated in kilobase pairs.
(**C**) SDS-PAGE analyses of fractions from glycerol gradient sedimentation of core histones and Mst77F. The proteins sediment at the top of the gradient, consistent with their unbound state.
(**D**) SDS-PAGE analyses of fractions from glycerol gradient co-sedimentation of APL and Mst77F. Sub-stoichiometric APL binds Mst77F and partially shifts its sedimentation profile towards the middle of the gradient.
(**E**) SDS-PAGE analyses of fractions from glycerol gradient co-sedimentation of APL, core histones and Mst77F. Sub-stoichiometric APL preferentially binds core histones and partially shifts their sedimentation profile towards the middle of the gradient.

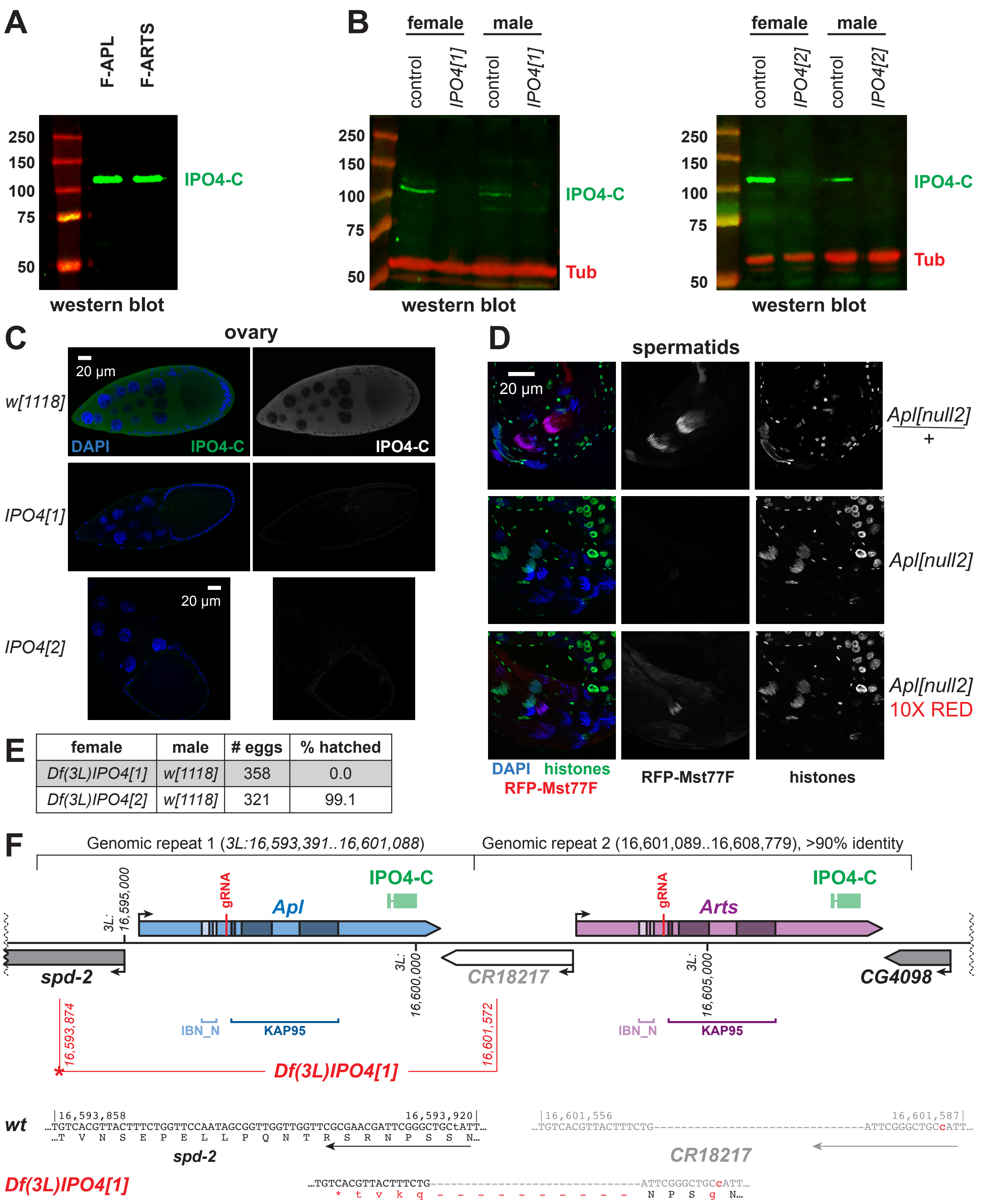

Suppl. Fig. S4. APL-mediated deposition of Mst77F into sperm chromatin during spermatogenesis *in vivo*.

(**A**) IPO4-C immunoblot analyses of recombinant APL and ARTS. ~10 ng each of purified recombinant FLAG-APL or FLAG-ARTS was loaded next to a mass marker. Sizes (kDa) are shown on the left. IPO4-C antibody recognizes both proteins (green).

(**B**) IPO4-C immunoblot analyses of mutant alleles. Protein lysates of whole adult females and males were analyzed by western. The amount of loaded material per lane is approximately equivalent to one fly. Green, IPO4-C antibody; red, tubulin (Tub) antibody. Molecular mass marker sizes (kDa) are shown on the left.

(**C**) Confocal images of *Drosophila* ovaries stained with IPO4-C antibody. Full-length ARTS protein that encompasses its C-terminus is strongly expressed in *w[1118]* (control) ovaries but cannot be readily detected in the homozygous mutants, Df(3L)IPO4[1] or Df(3L)IPO4[2]). Blue, DAPI staining of DNA; green or white, IPO4-C IF staining; scale bar, 20 µm.

(**D**) Defective loading of Mst77F in spermatids of the null mutant allele of *Apl* (16). The distribution of core histones was detected by IF staining, and the distribution of mRFP1-Mst77F transgenic protein was detected by auto-fluorescence and confocal microscopy, compare to **Fig. 4E**. Blue, DAPI staining of DNA; red or white, mRFP1-Mst77F; green or white, core histones; scale bar, 20 µm; 10X RED, 10-fold intensity increase for the red channel.

(**E**) Embryo hatching ratio as a measure of female fertility. Eggs laid by homozygous *Df(3L)IPO4[1]* females mated to *w[1118]* (control) males completely fail to hatch to larvae. In contrast, eggs laid by homozygous *Df(3L)IPO4[2]* females exhibit a normal hatch ratio, comparable to that for *w[1118]* (control) females (**Fig. 4C**).

(**F**) A frameshift mutation introduced in the 5'-end of *spd-2* by the *Df(3L)IPO4[1]* deficiency. *Apl – Arts* locus and all designations are as in **Fig. 4A**. Black typeface represents the *spd-2* sequence, whereas gray typeface represents the sequence of a homologous region in *CR18217*. Note that *spd-2* is transcribed (and translated) in the (-) orientation relative to the genome coordinates. Residues shown in lower-case, red typeface indicate amino acid substitutions introduced by *Df(3L)IPO[1]*.
